# Free DNA partially clarifies discrepancies between qPCR and the conventional phage quantification method

**DOI:** 10.1371/journal.pone.0313774

**Published:** 2024-12-03

**Authors:** Saar Van Overfelt, Hans Duyvejonck, Femke Baeke, Riet De Rycke, Maya Merabishvili, Stefan Vermeulen, Piet Cools, Mario Vaneechoutte, Els Van Mechelen

**Affiliations:** 1 Research Centre Health and Water Technology, University of Applied Sciences and Arts Ghent, Ghent, Belgium; 2 Faculty of Medicine and Health Sciences, Department of Diagnostic Sciences, Laboratory Bacteriology Research (LBR), Ghent University, Ghent, Belgium; 3 VIB BioImaging Core, Ghent, Belgium and VIB Center for Inflammation Research, VIB, Ghent, Belgium; 4 Department of Biomedical Molecular Biology, Ghent University, Ghent, Belgium; 5 Laboratory for Molecular and Cellular Technology (LabMCT), Burn Wound Center, Queen Astrid Military Hospital, Brussels, Belgium; Universidade de Lisboa Instituto Superior Tecnico, PORTUGAL

## Abstract

To use phages in a personalized therapy and industrial applications, an accurate quantification is needed. The gold standard method, namely the culture-based double agar overlay (DAO) method, provides an accurate estimate of the number of infectious phages but is laborious and time-intensive. Quantitative polymerase chain reaction (qPCR) can be used as a fast alternative but tends to overestimate the number of infectious phage particles. Here we describe the use of a DNase treatment before quantification of the *Staphylococcus aureus* phage ISP with qPCR to obtain a more accurate estimate of the number of infectious phage particles. We showed that DNase treatment results in a significant decrease of the concentration when measured with qPCR although for two out of three tested ISP phage stocks, there was still a significant difference with the DAO method. We also showed that the discrepancy between quantification with qPCR and the DAO method is dependent on the storage period of the phage stock, with a larger discrepancy for older stocks. Additionally, we used negative contrast immune electron microscopy to confirm the presence of DNA in the medium of the phage stock and the impact of the DNase treatment on the free DNA.

## Introduction

Bacteriophages, the viruses of bacteria, were discovered independently by Twort in 1915 and Félix d’Hérelle in 1917. D’Hérelle was the first to apply phage therapy (PT) in 1919 for the treatment of bacterial infections. This therapy was further developed and applied in the former Soviet Union and East European countries. In contrast it was being abandoned in Western Europe due to the rise of antibiotics. The emergence of the world wide spread of antimicrobial resistance (AMR) led to a renewed interest in PT in Western countries, where it is currently used in clinical trials and as personalized therapy to treat multidrug resistant bacterial infections. Nevertheless, many challenges, both practical and regulatory, still exist to implement phage therapy on a large scale in accordance with the standards of European and American legislation [1–4]. One of the important aspects of phage therapy is the dose that is administered during treatment, which can have a major impact on the treatment outcome [5–7]. In order to administer the correct dose to the patient, accurate quantification of the phages is needed. Proper titration of phages is also required in any industry where bacterial load or processing is important, for example, within the field of drinking water. Somatic coliphages are indicators of fecal/viral water pollution and have been introduced in water quality legislation as source tracking markers. Phages are being used for monitoring the efficiency of water treatment and purification in the Directive (EU) 2020/2184 on the quality of water intended for human consumption and in Regulation (EU) 2020/741 on minimum requirements for water reuse [8].

The gold standard to quantify phages is the double agar overlay (DAO) method. This method is based on the culturing of a bacterial host together with a phage, resulting in the formation of a countable number of plaques after successful phage infection [9]. However, many other techniques are currently used that detect either the phage particle as a whole, (part of) the phage genome or phage proteins [10]. Using, for example, quantitative PCR (qPCR) instead of the DAO method offers a number of advantages such as a better precision, a better agreement between different laboratories, a less laborious and time-consuming method and the possibility for high-throughput analyses [11,12]. Nevertheless, a major downside of qPCR is the inability to discriminate between infectious and non-infectious phages. This results in higher concentrations obtained by qPCR compared to the culture-based DAO method [11–15].

In a previous study, we described the development of a qPCR platform to quantify five phages in bacteriophage cocktail 2 (BFC2) in a fast and accurate manner [12]. The phages included in that therapeutic cocktail target clinical strains of three major bacterial pathogens, namely *Acinetobacter baumannii*, *Pseudomonas aeruginosa* and *Staphylococcus aureus* [16,17]. We observed that the difference between the concentration obtained with qPCR and the DAO method is phage-dependent and remains constant for different stocks of the same phage [12]. For the *S*. *aureus* phage ISP, the concentration obtained with qPCR was on average 7.27 ± 0.21 times higher compared to quantification with the DAO method, while a 67.41 ± 20.40 fold difference was observed for the *A*. *baumannii* phage Acibel007 [12]. Additionally, when phage stocks are quantified with qPCR after different storage periods, a constant concentration is observed (H. Duyvejonck, M. Vaneechoutte and E. Van Mechelen, unpublished data). When the same is done by using the DAO method, the concentration will decrease over time [18]. This led to the hypothesis that DNA is freely present in the storage medium or in noninfectious phage particles where it can be detected with qPCR. As a consequence, the number of infectious phage particles is overestimated by qPCR. The goal of this study was to verify this hypothesis in stocks of the *S*. *aureus* phage ISP.

## Materials and methods

### Experimental set up

**Fig 1:**
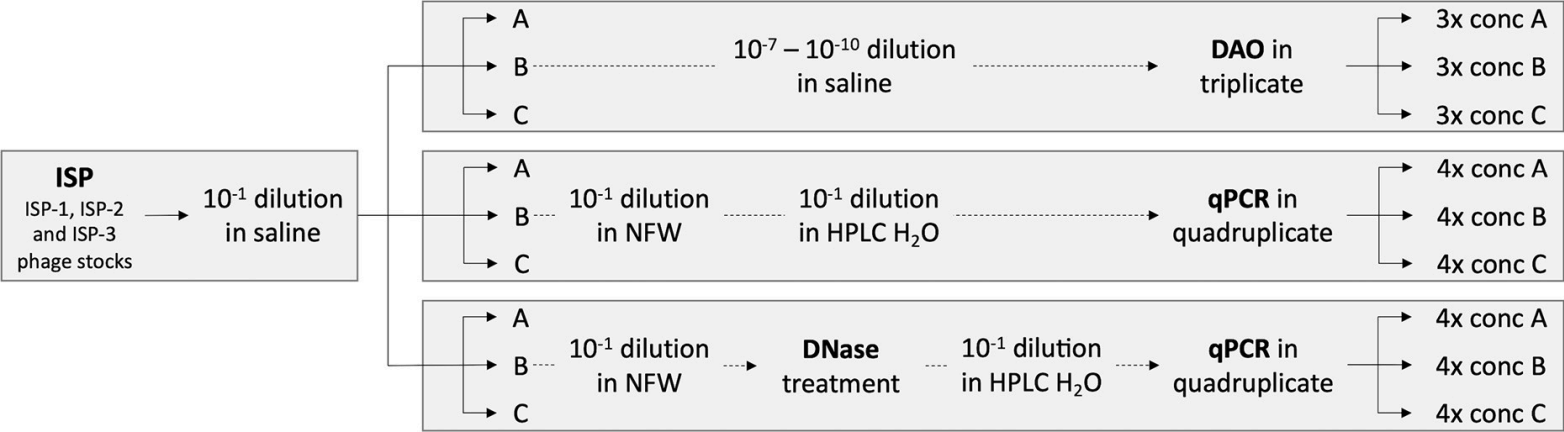
Experimental set up to determine the impact of DNase treatment of ISP phage stocks with different storage periods (ISP-1, ISP-2 and ISP-3) on the quantification with qPCR and the agreement between qPCR and DAO. NFW = Nuclease-free water.

To verify the presence of free DNA in phage stocks of the *S*. *aureus* phage ISP, a DNase treatment of the stock was performed prior to qPCR. The impact of this treatment on the concentration of the phage stocks was assessed by means of qPCR and evaluated as shown in Fig 1. The experiment was performed using three ISP phage stocks that had been stored at 4°C for different periods of time, namely 4.5 months (ISP-1), 8 months (ISP-2) and 16 months (ISP-3). A ratio of the concentrations was determined as follows. The concentration of the phage stock as obtained with qPCR, either before or after DNase treatment, was divided by the concentration of the phage stock as determined with DAO. The concentration of the DNase-treated stock was not determined with DAO since it was shown that DNase treatment led to the partial inactivation of the phages, resulting in a decreased concentration measured with DAO (Supplementary information). The entire experimental set-up as shown in Fig 1 was performed in triplicate with the ISP phage stock ISP-2. The results are presented as the averages of all measurements.

Additionally, negative contrast immune electron microscopy (NCIEM) was used to verify the presence of DNA in the phage stock and the impact of DNase treatment on the present DNA.

### Quantification by means of the double agar overlay assay

The phages were quantified as described previously using the DAO method [12,17]. Briefly, the bacterial host, *S*. *aureus* ATCC 6538, was grown aerobically overnight at 37°C on an LB agar slant after which a bacterial suspension was made in saline with a final concentration of 10^9^ cfu/ml. A tenfold dilution series of the phage stock (10^−4^ to 10^−11^) was prepared in saline and 1 ml of each dilution was mixed with 100 μl of the bacterial suspension and 3.5 ml LB broth (VWR, Leuven, Belgium) supplemented with 0.6% (w/v) Bacto agar (Becton Dickinson, Erembodegem, Belgium) in triplicate. Next, the 4.6 ml of this mixture was poured onto a 90 mm Petri dish containing an LB agar bottom layer (VWR) and incubated overnight at 37°C. After incubation, the number of plaques on the plates with distinguishable homogenous plaques were counted and the average concentration of the phage stock in plaque forming units per ml (pfu/ml) was calculated.

### Production of phage stocks with the double agar overlay assay

Bacteriophage stocks were prepared using the DAO method as described previously [12]. Briefly, the concentration of phages and bacteria resulting in a webbing pattern was first determined using the DAO method. Next, a large number of 90 mm Petri dishes, containing an LB agar layer, were prepared by pouring 3.5 ml of a mixture on the plate containing bacteria, phages and 0.6% (w/v) semi-solid LB agar, at the concentration that had been determined to result in a webbing pattern. The plates were incubated overnight at 32°C. Next, 500 μl chloroform (Sigma Aldrich, Steinheim, Germany) was added to the lid of the inverted plates, followed by incubation at 4°C for 1 h. Subsequently, the semi-solid agar layer was collected with a sterile L-shaped rod in sterile centrifuge tubes and centrifuged at 6000 *g* for 20 min. Then, the supernatant was aspirated with a 22G X 2” needle (Henke Sass Wolf, Tuttlingen, Germany) and filtered, consecutively with a 0.45 μm and a 0.22 μm filter (Merck, Overijse, Belgium). Finally, the phages were concentrated by high-speed centrifugation (35 000 *g*, 1 h). The pellet was resuspended overnight in saline and stored in a 15 ml Falcon tube at 4°C until use.

### DNA extraction

Phage DNA was extracted using the PureLink Viral RNA/DNA Mini Kit (Invitrogen, Carlsbad, California) according to the manufacturer’s instructions. The extraction was started from 200 μl of the phage stock and the DNA was eluted in 200 μl nuclease-free water (NFW) to avoid concentrating the DNA compared to the phage stock.

### DNA yield and purity

The yield of the extracted DNA was determined using the Qubit dsDNA HS Assay Kit (ThermoFisher Scientific, Merelbeke, Belgium). The purity was expressed as the ratio between the absorbance at 260 nm and 280 nm as measured with the Denovix DS-11+ spectrophotometer (Denovix, Wilmington, USA).

### DNase treatment

Phage stocks were treated with a DNase prior to qPCR to remove DNA that is freely present in the stock. The phage stock was diluted in saline until a concentration of 10^10^ pfu/ml, based on quantification with DAO, was reached, followed by a 1/10 dilution in NFW to reduce the salt concentration. The treatment was performed as follows: to a volume of 190 μl 10^9^ pfu/ml phage stock, 40 U RQ1 RNase-Free DNase (1 U/μl, Promega, Leiden, The Netherlands), 30 μl 10x reaction buffer and 40 μl NFW were added. This mixture of 300 μl was incubated at 37°C for 30 min after which 30 μl stop solution was added followed by an incubation at 65°C for 10 min. As a control of the DNase activity, the treatment was also performed using 190 μl of a DNA extract of the ISP phage stock.

### Quantification of phage stocks with quantitative PCR (qPCR)

According to MIQE guidelines the phage stocks were quantified with qPCR before and after DNase treatment, as described previously [12]. The phage stocks were tenfold diluted in HPLC-grade water after which 2 μl was added to 8 μl of the reaction mixture, which contained LightCycler 480 High Resolution Melting Master Mix (Roche, Brussels, Belgium), 0.2 μM of both the forward and reverse primer and 3 mM MgCl_2_. The sequence of the forward primer was 5’ CCGGCTTGACTCTCATTCCA3’ and the sequence of the reverse primer was 5’ AGCTACAACCGAGCAGTTAGA3’ [12]. Each stock was quantified in quadruplicate. The qPCR reaction was performed on a LightCycler 480 (Roche) using the following steps: pre-incubation at 95°C for 5 min, 45 amplification cycles consisting of denaturation at 95°C for 10 s, primer annealing at 60°C for 15 s and elongation at 72°C for 15 s. A calibration curve was constructed based on tenfold serial dilutions of a DNA extract of the ISP phage stock. First, the DNA concentration of the extract was measured with the Denovix DS-11+ and converted from ng/μl to chr/ml based on the GC-content and the genome size of ISP (NC_047720.1). Next, a calibration curve was constructed with the logarithmic concentration (chr/ml) on the x-axis and the obtained Cq value on the y-axis.

To verify the specificity of the amplification, melting curve analysis was performed, using the following protocol: 95°C for 1 min, 40°C for 1 min, 60°C for 1 s and a continuous increase from 60°C to 97°C with a ramp rate of 0.04°C/s and 15 readings/°C. A melting temperature of the amplicon of 75.98°C was considered specific for ISP [12].

### Negative contrast immune electron microscopy (NCIEM)

To visually confirm the presence of DNA in the ISP phage stock, NCIEM was performed on an untreated stock. Additionally, a DNase-treated ISP phage stock, without heat-inactivation of the DNase, was visualized to confirm the impact of DNase treatment on the DNA in the stock. Ni-grids were formvar/carbon-coated and given glow discharge for 10 sec followed by the adsorption of the sample on the grid for 1 min. The grids were then washed 3 times in Milli-Q water and incubated in 10 mM PBS for 5 min followed by 30 min incubation in blocking solution (905.001, Aurion, Wageningen, The Netherlands) and washed five times for 5 min in incubation buffer (900.099, Aurion). Next, the grids were incubated in a 1/50 or 1/200 dilution of the primary antibody against dsDNA (HYB331-01, Santa Cruz Biotechnology, Heidelberg, Germany) for 1–2 h, followed by five washing for 5 min in incubation buffer. This was followed by incubation with the AffiniPure rabbit anti-mouse IgG bridging antibody (315-005-003, Jackson ImmunoResearch, West Grove, Pennsylvania) for 20 min and five washes of 5 min each in incubation buffer. Then, the protein A conjugated with a 5 nm gold particle (Cell Microscopy Core, University Medical Center Utrecht, Utrecht, The Netherlands) in incubation buffer was added for 30 min followed by two washes of 5 min in incubation buffer, three washes of 5 min in PBS and 5 washes of 2 min in Milli-Q water. Finally, the grids were negatively stained in a 1% uranyl acetate solution for 10 sec and allowed to air dry for 24 h. Visualization was performed with a JEM 1400plus transmission electron microscope (JEOL, Tokyo, Japan) at 80 kV. Artefacts possibly introduced by the immune labeling and background labeling were controlled by performing only the negative staining and the immune labeling without the primary antibody, respectively.

### Statistical methods

Statistical analyses were performed in R v4.1.2 and graphs were made using Excel and the package ggplot2 v3.3.5 in R. Significant differences between the ratio of concentrations before and after DNase treatment were tested in R using a paired student t-test in case the data were normally distributed or the Wilcoxon Signed Rank test if the data did not follow a normal distribution. To perform multiple pairwise comparisons between all obtained concentrations, Model 1 ANOVA was used followed by post-hoc comparisons with corrected p-values according to the Tukey procedure. P-values below 0.05 were considered significant.

## Results

### Impact of DNase treatment on concentration of phage stocks

**Fig 2:**
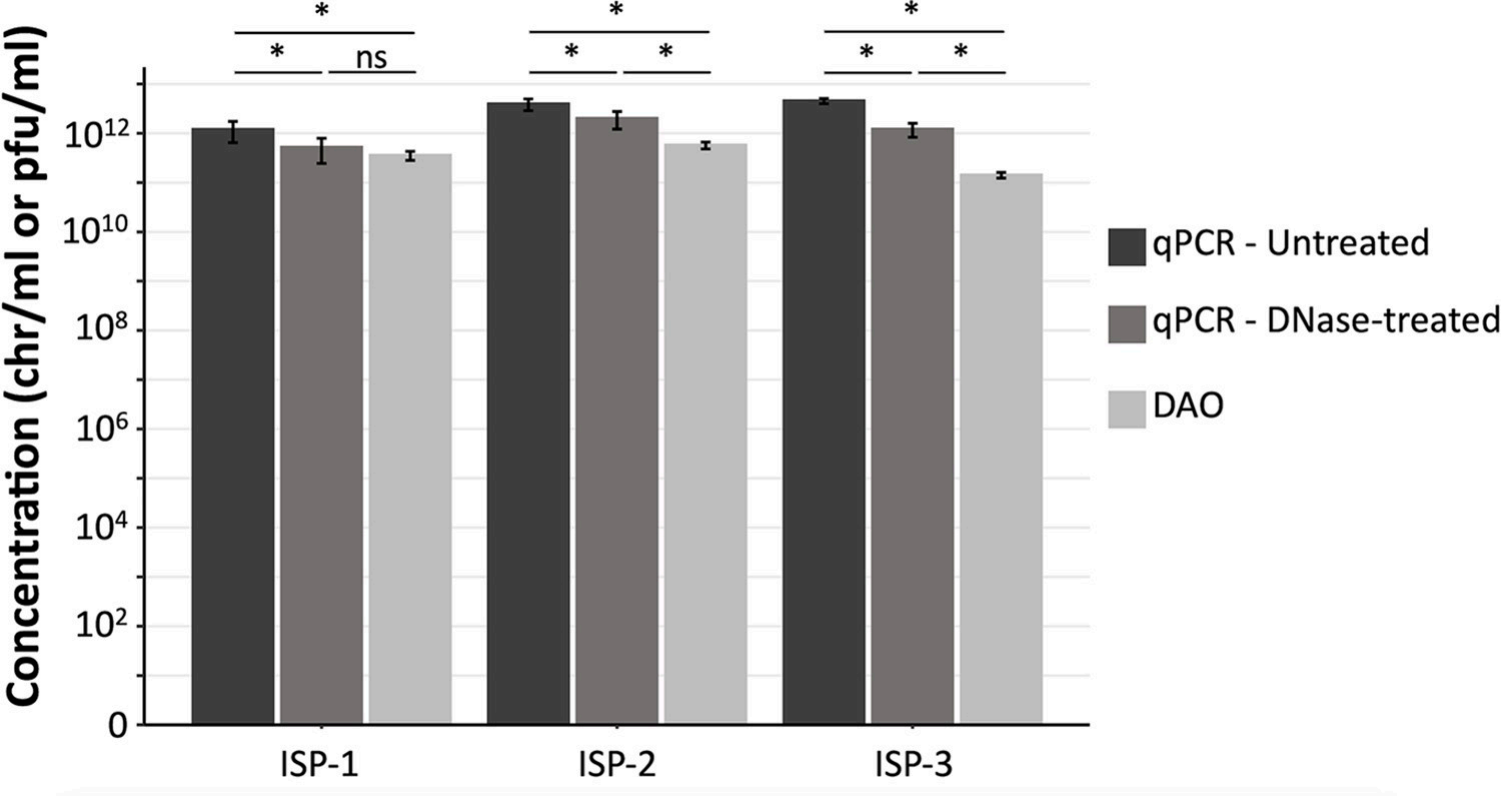
Average concentration of the ISP phage stocks obtained with qPCR, before and after DNase treatment, and with the DAO method before the DNase treatment. The error bars indicate the standard deviation. Significant differences were calculated with a model 1 ANOVA. * = p < 0.05, ns = non-significant (p > 0.05).

**Fig 3:**
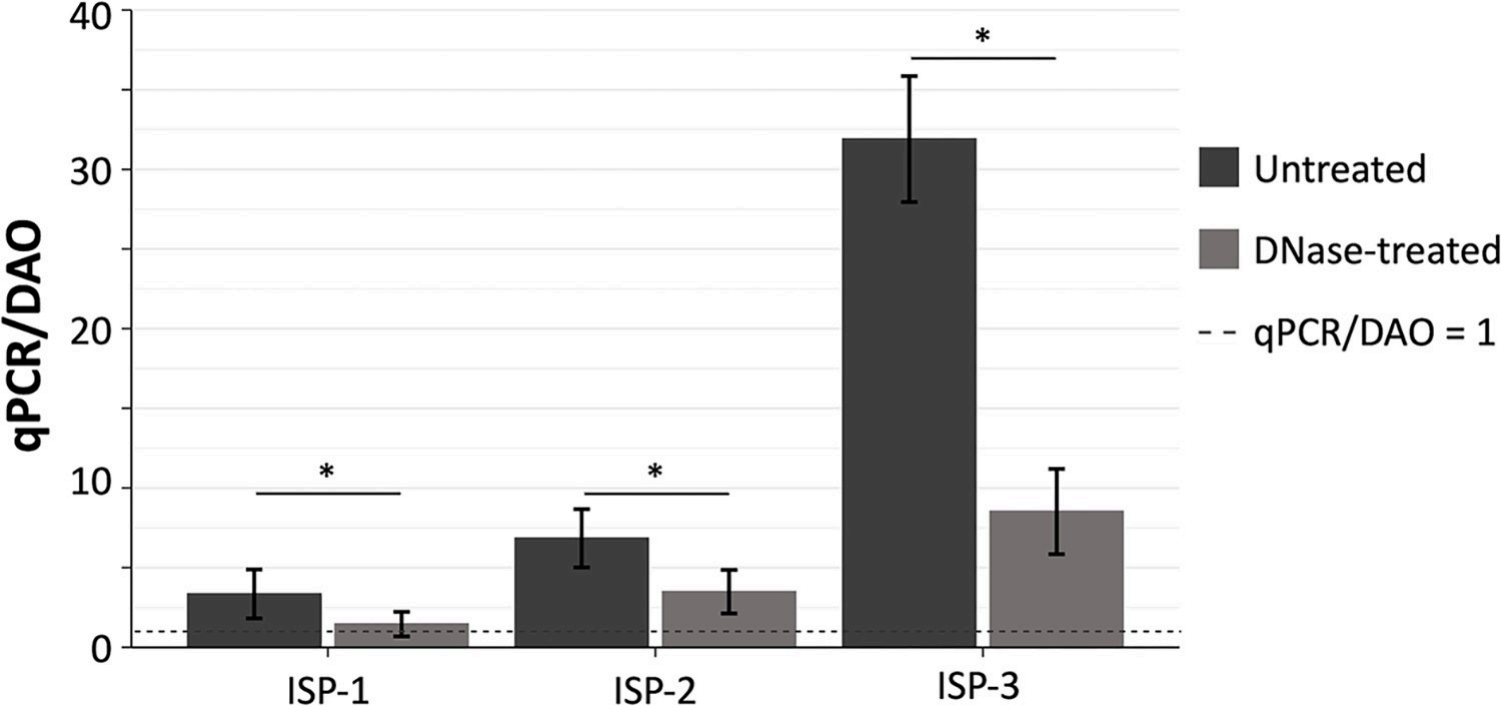
Average ratio of the concentrations obtained with qPCR, before and after DNase treatment, and with DAO before DNase treatment of the ISP phage stocks. The error bars indicate the standard deviation. Significant differences were calculated with a Student t-test. * = p < 0.05.

The ISP phage stock ISP-1 had an average concentration of 3.56 ± 0.74*10^11^ pfu/ml according to the DAO method and 1.19 ± 0.55*10^12^ chr/ml according to qPCR (Fig 2). This resulted in a qPCR/DAO ratio of 3.35 ± 1.53 (Fig 3). Pretreatment of the phage stock with a DNase resulted in a significant decrease to a concentration of 5.19 ± 2.74*10^11^ chr/ml (p < 0.001) as measured with qPCR, corresponding to a qPCR/DAO ratio of 1.46 ± 0.77. This concentration was not significantly different from the concentration obtained with the DAO method (p = 0.59).

A comparable decrease in concentration after DNase treatment was obtained for the ISP phage stock ISP-2 (Figs 2 and 3). The concentration obtained using the DAO method equaled 5.74 ± 0.92*10^11^ pfu/ml. When measured with qPCR before and after a DNase pretreatment, the concentration equaled 3.93 ± 1.05*10^12^ chr/ml and 2.00 ± 0.78*10^12^ chr/ml, respectively. After DNase treatment, with a qPCR/DAO ratio of 3.49 ± 1.37, the concentration obtained with qPCR was still significantly different from the concentration obtained with the DAO method (p < 0.001).

The phage stock that was stored for the longest period (i.e., 16 months, ISP-3) had a concentration of 1.42 ± 0.19*10^11^ pfu/ml when measured with DAO and this increased 31.9 ± 3.95 fold when measured with qPCR, resulting in a concentration of 4.54 ± 0.56*10^12^ chr/ml (Figs 2 and 3). When DNase treatment was performed prior to qPCR, the obtained concentration decreased significantly to 1.21 ± 0.38*10^12^ chr/ml (p < 0.001), which still represents an 8.52 ± 2.67 fold increase compared to DAO. The obtained concentration after DNase treatment remained significantly different from the concentration obtained with DAO (p < 0.001).

### Analysis of DNase treatment by NCIEM

**Fig 4:**
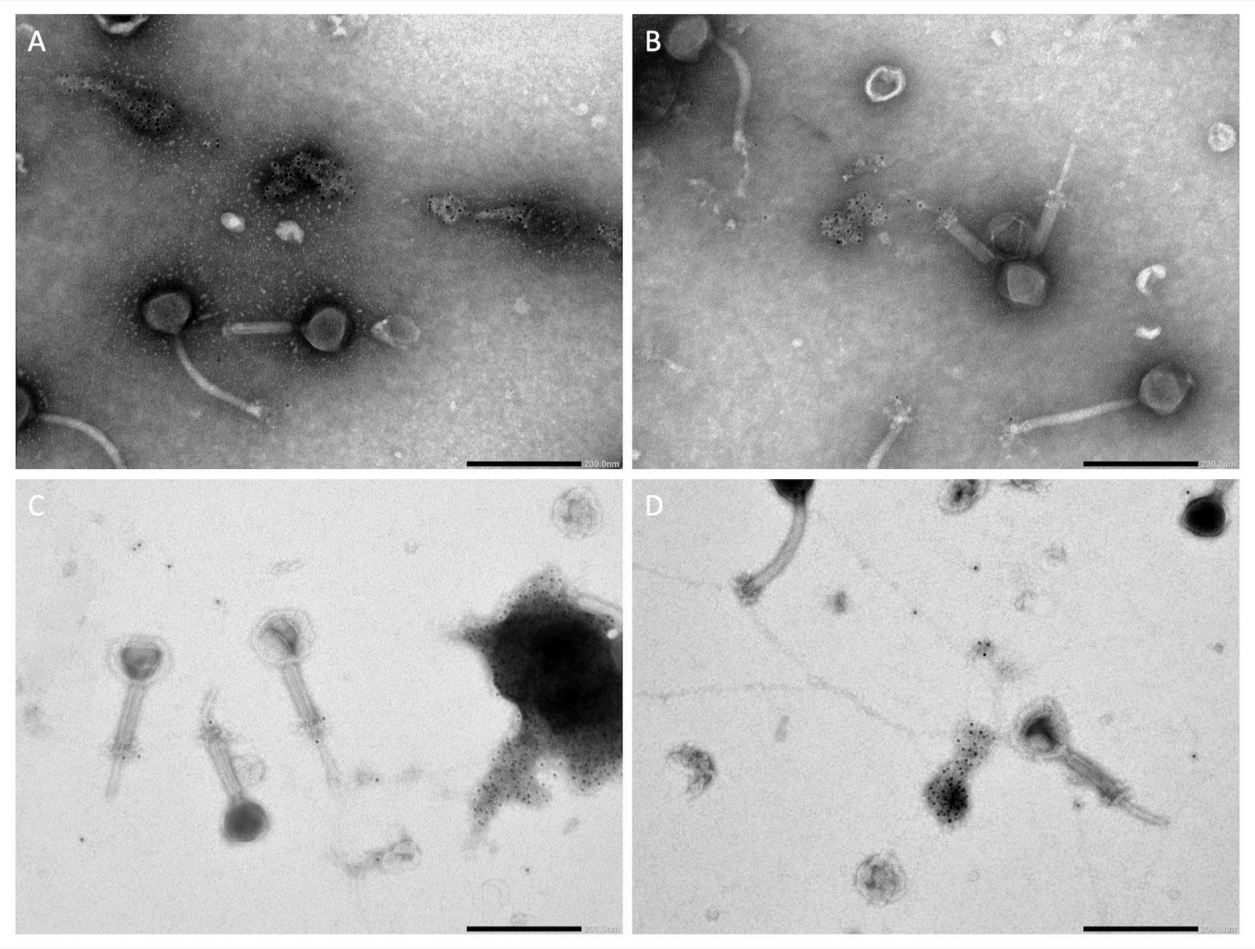
Visualization of DNA in an untreated ISP phage stock by immunogold labelling. DNA is visualized by gold particles (•). Scale bar = 200 nm.

To validate the results found with qPCR, ISP phage stock ISP-2 was visualized with NCIEM using an antibody against dsDNA. At the time of visualization, the stock was 1 year, 4 months and 18 days old. The presence of free DNA in the ISP phage stock was confirmed by the visualization of gold particles as shown in Fig 4. Gold particles, indicating the presence of DNA, are detected separate from the phage particles in the medium. DNA formed clusters (Fig 4A and 4B) and threads between different ISP phages (Fig 4C and 4D). Sequencing and qPCR specific for *S*. *aureus* confirmed that the DNA in the phage stock originated predominantly from ISP and not from its host (data not shown). Therefore, we can conclude that the free DNA originated from ISP and contributed to the concentration measured with qPCR. When visualizing ISP-2 after DNase treatment, but before heat-inactivation, a decreased amount of free DNA was observed in the stock, although the phage structure was greatly impaired.

In Fig 4, gold particles are also visible on the baseplate, the tail fibers and the tail tube of some phages, suggesting that DNA was also present on these structures, but visualization without the primary antibody showed that this was non-specific background labeling.

## Discussion

In a previous study, we observed a phage-dependent difference in estimated concentrations between qPCR and DAO quantification methods [12]. We hypothesized that this difference might be due to free DNA in the phage stock, originating from damaged phages. This free DNA remains stably present in the phage stock, thereby leading to overestimation of the number of infective phages with qPCR, a problem not encountered with the DAO method. To test this hypothesis, we first investigated the impact of DNase treatment on the integrity of the phage particles (Supplementary information). Next, we showed that DNase pretreatment of the ISP phage stocks followed by quantification with qPCR resulted in a significantly decreased concentration compared to quantification with direct qPCR. For the ISP phage stock ISP-1, the qPCR/DAO ratio decreased from 3.35 ± 1.53 to 1.46 ± 0.77, resulting in a non-significant difference between quantification with qPCR after DNase treatment and DAO. This initial difference between qPCR and the DAO method in fresh phage stocks can be caused during the propagation procedure by the release of DNA from degraded phages into the medium. Chloroform is often used to release phages from infected cells [19] but this inactivates approximately 30% of the tailed phages [20]. Additionally, the presence of bacterial debris in the phage stock increases when chloroform is used because it lyses the remaining bacterial cells [19]. This debris can then be recognized by the phage as an intact bacterial cell thereby activating the phage to eject its DNA. The use of polyethylene glycol precipitation and cesium chloride density gradients to concentrate and purify the phage stock are also known to negatively impact the activity of the phages [21]. In this case, the DNA is released in the medium, making it easily accessible for the DNase to be degraded.

However, after treatment of the ISP-2 and ISP-3 phage stocks with a DNase, the concentration obtained with qPCR was still significantly higher than the concentration determined with DAO. We also observed that the discrepancy between quantification with the DAO method and qPCR increased when the phage stocks were stored for a longer period, even after DNase pretreatment of the stock. During the storage period, phages can lose infectivity when the tail is lost or damaged while the capsid of most of these phages remains intact [22]. The degree of this phage inactivation depends on the used storage medium and the initial phage concentration, resulting in either a rapid inactivation or only minor losses in activity over time [18,23]. Since the DNA is still inside the intact capsid, it might be inaccessible for the DNase to be degraded, resulting in a larger discrepancy between qPCR after DNase treatment and quantification with the DAO method.

A comparable qPCR-determined decrease of the concentration after DNase treatment, was observed for other phages [13,15,24]. Peng, Nguyen and Gosh described comparable results for the quantification of phages M13 and T7. They found that, for an M13 phage stock, the concentration obtained with qPCR was 3.3-fold higher than with DAO. Pretreatment of the phage stock with the DNase resulted in a 2.5-fold decreased concentration when measured with qPCR. Additionally, they found significant differences between quantification of a T7 phage stock with DAO and a DNase-pretreated stock with qPCR [13]. Edelman and Barletta quantified the contribution of uncoated DNA to the qPCR signal for phage lambda. Their results showed that quantification of an untreated phage stock resulted in approximately a 1 log increased concentration compared to a DNase pretreated stock [15]. Digital droplet PCR (ddPCR) was used by Morella and colleagues to quantify the *Pseudomonas syringae* phages FRS and SHL. ddPCR resulted in a two-three orders of magnitude increased concentration compared to the traditional DAO method. After DNase and proteinase K treatment, they could still detect a two to three orders of magnitude increased concentration with ddPCR although the obtained concentration after enzyme treatment was three to four times lower compared to no treatment [24].

Next to a DNase treatment, the presence of free DNA in the ISP phage stock and the impact of the DNase treatment were investigated by using NCIEM with antibodies against dsDNA. DNA could clearly be visualized outside the ISP phage particles in the form of clusters and threads and after DNase treatment, the amount of free DNA was reduced. This confirmed the results obtained by applying a DNase treatment prior to quantification with qPCR and our hypothesis that free DNA in the ISP phage stock results in an overestimation of the concentration obtained with qPCR.

In conclusion, we showed that, for the *S*. *aureus* phage ISP, free DNA is a main cause of the difference in quantification with qPCR and DAO and the pretreatment of the phage stock with a DNase results in a better agreement between both methods. Nevertheless, it remains questionable if this is also true for the other phages in BFC2 and it might be necessary to optimize the DNase treatment for each phage separately, since the observed qPCR/DAO ratio differed largely depending on the phage [12].

## Supporting information

S1 Fig Qubit-based quantification of the DNA present after different steps (P1—P7) of the DNase treatment (A) and visualization of free DNA present after steps P1—P7 of the DNase treatment (B). The error bars indicate the standard deviation. Model 1 ANOVA: * = p < 0.05, ns = non-significant (p > 0.05). DNA gel electrophoresis on a 0.6% (w/v) agarose gel (120 V, 100 min). Samples were: P1 = untreated, P2 = incubation at 37°C, P3 = addition of reaction buffer and incubation at 37°C, P4 = incubation at 37°C and 65°C, P5 = addition of reaction buffer and incubation at 37°C and 65°C, P6 = addition of reaction buffer, incubation at 37°C, addition of stop buffer and incubation at 65°C, P7 = addition of 40 U DNase and reaction buffer, incubation at 37°C, addition of stop buffer and incubation at 65°C.
(DOCX)

S2 Fig DNA in an ISP phage stock after heat incubation (65°C, 10 min), visualized by immunogold labelling. DNA is visualized by gold particles (•). A) Scale bar = 500 nm, B) Scale bar = 200 nm.
(DOCX)
