# Supplementary material for: Free DNA partially clarifies discrepancies between qPCR and the conventional phage quantification method
Source: PLoS One. 2024 Dec 3;19(12):e0313774. doi: 10.1371/journal.pone.0313774 (PMC11614264; doi:10.1371/journal.pone.0313774)
Supplement: S1 Fig — Qubit-based quantification of the DNA present after different steps (P1—P7) of the DNase treatment (A) and visualization of free DNA present after steps P1—P7 of the DNase treatment (B). The error bars indicate the standard deviation. Model 1 ANOVA: * = p < 0.05, ns = non-significant (p > 0.05). DNA gel electrophoresis on a 0.6% (w/v) agarose gel (120 V, 100 min). Samples were: P1 = untreated, P2 = incubation at 37°C, P3 = addition of reaction buffer and incubation at 37°C, P4 = incubation at 37°C and 65°C, P5 = addition of reaction buffer and incubation at 37°C and 65°C, P6 = addition of reaction buffer, incubation at 37°C, addition of stop buffer and incubation at 65°C, P7 = addition of 40 U DNase and reaction buffer, incubation at 37°C, addition of stop buffer and incubation at 65°C. (DOCX) [file pone.0313774.s001.docx]

#### **Impact of DNase treatment on phage integrity**

After optimization of the DNase treatment to a final DNase concentration of 40 U, the impact of each step in the DNase treatment on the integrity of the phage particles was evaluated by agarose gel electrophoresis and DNA quantification with Qubit. The different components of the treatment, which were: addition of reaction buffer, incubation at 37 °C for 30 min, addition of stop buffer and incubation at 65 °C for 10 min were performed in a stepwise fashion, starting with incubation at 37 °C without the addition of any buffer.

#### **Gel electrophoresis**

To evaluate the impact of each step during the DNase treatment for the integrity of the ISP phage particles, the treated phage stock was loaded on an agarose gel to visually inspect the release of DNA from phage particles by the occurrence of a DNA band or smear. A total of 20 µl of each sample was mixed with 5 µl of 5x loading dye and loaded onto the gel. The gel was prepared with 1x TAE, supplemented with ethidium bromide and the electrophoresis was performed in 0.5x TAE buffer. The DNA was visualized by illumination with UV at a wavelength of 302 nm using the Gel Doc XR+ System and the accompanying Image Lab software (Bio-Rad, Brussels, Belgium).

#### **Statistical methods**

Statistical analyses were performed in R v4.1.2 and graphs were made using Excel. Significant differences between the DNA concentration of the untreated ISP phage stock and the DNA concentration after performing each component of the DNase treatment were tested in R using Model 1 ANOVA followed by post-hoc comparisons with corrected p-values according to the Tukey procedure. P-values below 0.05 were considered significant.

### **Results**

#### **Impact of DNase treatment on the integrity of phage particles**

When performing DAO quantification before and after DNase treatment, the concentration decreased from 4.27 ± 0.19*10^10^ pfu/ml and 1.86 ± 0.47*10^6^ pfu/ml, respectively. We hypothesized that the phage particles were (partially) degraded because of the treatment. We therefore investigated during which step the degradation of the phages occurred. The effect on the phage particles was observed by using DNA quantification with Qubit and gel electrophoresis (Fig S1). We investigated the degradation of the phages by measuring the DNA concentration with Qubit. When the phage particles are being degraded, it is expected that the DNA is released from the phage particles, resulting in an increased DNA concentration when measured with Qubit. Gel electrophoresis was used to obtain a visual confirmation of phage DNA being released after degradation.

**S1 Fig. Qubit-based quantification of the DNA present after different steps (P1 - P7) of the DNase treatment (A) and visualization of free DNA present after steps P1 - P7 of the DNase treatment (B).** The error bars indicate the standard deviation. Model 1 ANOVA: * = p < 0.05, ns = non-significant (p > 0.05). DNA gel electrophoresis on a 0.6 % (w/v) agarose gel (120 V, 100 min). Samples were: P1 = untreated, P2 = incubation at 37 °C, P3 = addition of reaction buffer and incubation at 37 °C, P4 = incubation at 37 °C and 65 °C, P5 = addition of reaction buffer and incubation at 37 °C and 65 °C, P6 = addition of reaction buffer, incubation at 37 °C, addition of stop buffer and incubation at 65 °C, P7 = addition of 40 U DNase and reaction buffer, incubation at 37 °C, addition of stop buffer and incubation at 65 °C.

Quantification with Qubit showed a minor, non-significant increase in the DNA concentration when the phage particles were incubated at 37 °C, both in the absence (P2, p = 0.97) and presence of the reaction buffer (P3, p = 0.92) compared to the untreated phage particles (P1). Additionally, there was no difference visible on the agarose gel between the untreated phage particles and the particles that underwent these treatments. From these results we could conclude that the phage particles remained largely intact during this first incubation at 37 °C, even in the presence of the reaction buffer.

Incubation at 65 °C (P4, p < 0.001) resulted in a significant increase in DNA concentration, and when the reaction buffer (P5, p < 0.001) and stop solution (P6, p < 0.001) were added, an additional major increase was observed compared to the untreated phages. When the phages were incubated at 37 °C and 65 °C without the addition of any buffer, no band was observed on the agarose gel, but when one or both buffers where added, a fragment with a length of approximately 48.5 kb was clearly visible. In conclusion, the degradation of the phage particles, resulting in the release of the phage genome, appeared to occur mainly during the incubation at 65 °C in the presence of the reaction buffer and stop solution.
