# Supplementary material for: Free DNA partially clarifies discrepancies between qPCR and the conventional phage quantification method
Source: PLoS One. 2024 Dec 3;19(12):e0313774. doi: 10.1371/journal.pone.0313774 (PMC11614264; doi:10.1371/journal.pone.0313774)
Supplement: S2 Fig — DNA is visualized by gold particles (•). A) Scale bar = 500 nm, B) Scale bar = 200 nm. (DOCX) [file pone.0313774.s002.docx]

#### **Negative contrast immune electron microscopy (NCIEM)**

To confirm the impact of heat incubation on the phage particles during the DNase treatment, an untreated and a heat-treated (65 °C, 10 min) ISP phage stock were studied with NCIEM as described before.

This was confirmed by using NCIEM in the presence of dsDNA antibodies (Fig S2). A large number of thick DNA stretches were visible in the storage medium, which are not visible when the untreated ISP phage stock is visualized (Fig 4).

**S2 Fig. DNA in an ISP phage stock after heat incubation (65 °C, 10 min), visualized by immunogold labelling. DNA is visualized by gold particles (•)**. A) Scale bar = 500 nm, B) Scale bar = 200 nm.

The release of phage DNA into the storage medium during incubation at 65 °C was not considered a problem for the applicability of the DNase treatment since the DNase gets inactivated at this point and cannot degrade DNA originating from intact, infectious phage particles. As such, DNase treatment will not influence DNA quantification by means of qPCR. For qPCR, phage particles are degraded anyway, during DNA-extraction.
